# Posterior Mitral Leaflet (PML) Perforation due to Infective Endocarditis (IE) Complicated with Cerebral Infarction

**DOI:** 10.7759/cureus.36491

**Published:** 2023-03-21

**Authors:** Hideki Sasaki, Yukihide Numata, Jien Saito, Yoshiaki Sone, Miki Asano

**Affiliations:** 1 Cardiovascular Surgery, Nagoya City University East Medical Center, Nagoya, JPN

**Keywords:** autologous pericardium, brain infarction, mitral valve perforation, mitral valve regurgitation, infective endocarditis

## Abstract

A 64-year-old man on dialysis presented to the emergency department with a fever and chills. Transthoracic echocardiography (TTE) showed small vegetation on the posterior mitral leaflet (PML). Antibiotic therapy was initiated. Two weeks later, right hemiparesis occurred. MRI of the head showed occlusion of the left middle cerebral artery, which suggested an embolism derived from the vegetation. The patient was then referred to the department of cardiovascular surgery. Transesophageal echocardiography (TEE) revealed perforation of the PML and severe mitral regurgitation (MR). The patient underwent mitral valve repair. The postoperative course was uneventful, and the patient was discharged after six weeks of antibiotic treatment. A fresh autologous pericardium is the material of choice to repair the valve.

## Introduction

*Staphylococcus aureus* is usually found in the skin and nasal cavities. The skin is known to harbor a diverse community of indigenous microorganisms, with notable representatives being* Staphylococcus epidermidis*, *Propionibacterium acnes*, and *Staphylococcus aureus*. Among these, *Staphylococcus aureus* is typically regarded as commensal, i.e., non-pathogenic, albeit with the potential to inflict serious complications in patients with underlying medical conditions. Infective endocarditis (IE) is a devastating disease caused by *Staphylococcus aureus*. IE occurs in approximately 3-10/100,000 people [1]. Its predisposing risk factors include intravenous drug use, valve prolapse, prosthetic valve replacement, intracardiac defects, and hemodialysis. Transthoracic echocardiography (TTE) may occasionally fail to detect early-stage small vegetation, rendering transesophageal echocardiography (TEE) a more effective diagnostic modality for detecting small lesions that TTE is incapable of visualizing. Following accurate diagnosis, the administration of antibiotics is the initial course of action. Nevertheless, in cases where patients exhibit recalcitrant heart failure or an embolism, surgical intervention is recommended [2]. Herein, we present a case of mitral valve perforation complicated by cerebral infarction.

## Case presentation

A 64-year-old man undergoing hemodialysis complained of two days of high fever and chills. CT revealed edema around the appendix. Although the patient was admitted to a regional hospital with suspected appendicitis, this was eventually ruled out. Upon admission, the laboratory results revealed a leukocyte count of 5,000/μL, hemoglobin concentration of 8.7 g/dL, and platelet count of 183,000/μL. Additionally, the patient exhibited an elevated blood urea nitrogen level of 55.0 mg/dL and a serum creatinine level of 11.01 mg/dL. Aspartate aminotransferase and alanine aminotransferase levels were measured at 19 U/L and 2 U/L, respectively, while C-reactive protein was elevated at 1.57 mg/dL. Furthermore, the patient's B-type natriuretic peptide level was found to be 3,876.3 pg/ml.

**Figure 1:**
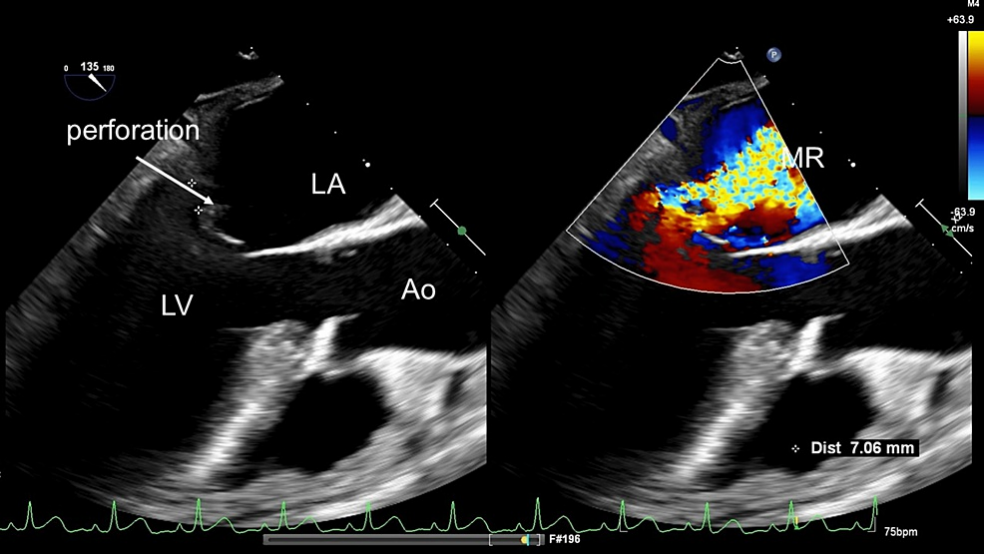
Preoperative TEE Preoperative TEE showed perforation (✜) of the PML TEE: Transesophageal echocardiography; MR: Mitral regurgitation; LV: Left ventricle; LA: Left atrium; Ao; Aorta; PML: Posterior mitral leaflet

Meropenem was started empirically because the patient was on dialysis and was vulnerable to infection. Repeated blood cultures tested positive for methicillin-susceptible *Staphylococcus aureus*. Sustained detection of *Staphylococcus aureus* in sequential blood cultures necessitated a comprehensive antibiotic sensitivity analysis, which revealed resistance to Penicillin G, and a minimum inhibitory concentration (MIC) of four to cefazoline. Given the development of central nervous system complications secondary to vegetation, we sought the expert opinion of the infection control team, who recommended ceftriaxone as the optimal therapeutic strategy. Accordingly, a daily dose of 6 g ceftriaxone was administered to the patient. The second CT revealed pericardial effusion, interlobular septal thickening, and pulmonary edema. Heart failure was suspected, and the patient was referred to the cardiology department. TTE revealed a small vegetation on the posterior mitral leaflet (PML) and mitral regurgitation (MR), which led to the diagnosis of IE. Two weeks later, the patient complained of aphasia and right hemiplegia. MRI of the head revealed occlusion of the left middle cerebral artery, probably due to vegetation. The patient was then referred to the department of cardiovascular surgery. TEE revealed a perforation of the middle segment of the PML and severe MR (Figure 1).

**Figure 2:**
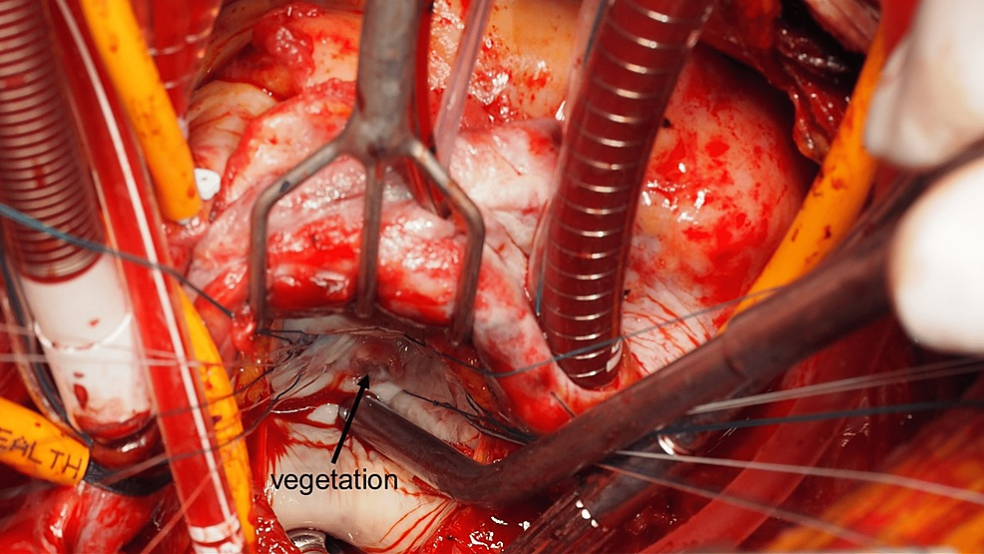
Intraoperative findings Vegetation and perforation on the PML PML: Posterior mitral leaflet

**Figure 3:**
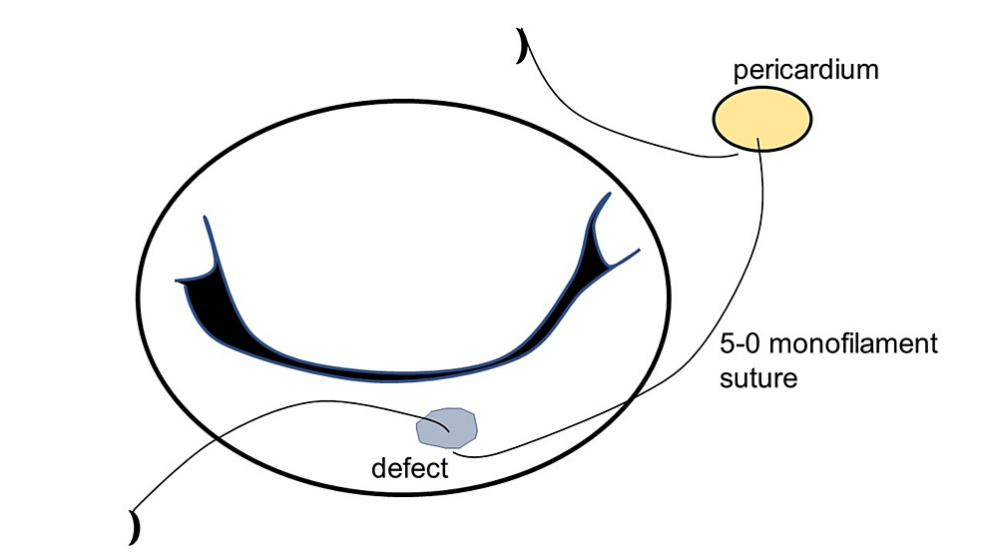
The autologous pericardial patch repair The autologous pericardial patch repair using 5-0 monofilament sutures. The patch is larger than the defect.

**Figure 4:**
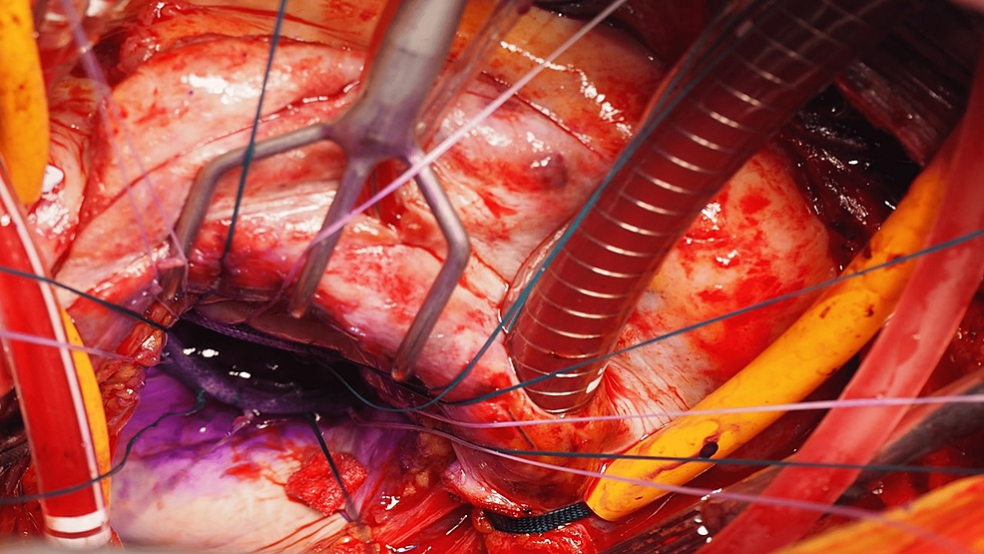
Mitral valve repair using autologous pericardium and annuloplasty ring A saline test showed no MR. MR: Mitral regurgitation

Under general anesthesia, cardiopulmonary bypass (CPB) was established with ascending aortic perfusion and vena cava drainage. After aortic cross-clamping, the mitral valve was inspected via left atriotomy. Vegetation and perforations (5-7 mm in diameter) were detected in the clear zone of the middle segment of the PML (Figure 2). After radical debridement, the diameter of the defect was 10-12 mm. An autologous pericardium was harvested and trimmed into a 20 × 22 mm oval shape. The defect was closed using 5-0 monofilament sutures (Figure 3). A prosthetic valvular ring (Carpentier Edwards Physio II ring, Edwards Life Sciences, Irvine, USA) was attached to the mitral annulus. A saline test revealed no MR (Figure 4). The patient was successfully weaned off CPB and transferred to the ICU. The patient awoke six hours later and was weaned off the ventilator. The postoperative course was uneventful, with no aggravation of hemiplegia. Throughout the patient's perioperative course, cardiac rhythm remained in sinus rhythm, with no episodes of paroxysmal atrial fibrillation detected on the electrocardiogram monitor.The patient was discharged without complications after six weeks of antibiotic treatment.

## Discussion

Once IE is diagnosed, antibiotic therapy is administered. However, uncontrollable heart failure and large vegetations (> 10 mm) are indications for prompt surgery. As in our case, surgery should be performed immediately after an embolism occurs. Surgeons generally have two options: valve repair or valve replacement. In this case, the vegetation was located in the clear zone of the middle segment of the PML and did not invade the annulus. Hence, we concluded that valve repair was feasible. Valve repair using autologous tissues, such as the pericardium, is the most desirable procedure. Previous studies have reported the use of autologous pericardium to repair valves [3,4]. Although autologous tissue is a good option for compensating defective areas, its durability is a problem. Moreover, whether fresh or glutaraldehyde-treated pericardium should be used is also controversial [5]. The pericardium may lose pliability or become calcified over time.

When encountering a perforation, surgeons must consider two points: One is to apply a pericardium larger than the defect after debridement; the second is to avoid the purse-string effect. If an equally sized pericardium is applied to the defect, perforation may recur due to cutting of the edematous leaflet.

Regarding the suture method, interrupted sutures are better than continuous sutures as they can minimize the potential of the purse-string effect. When a continuous suture is used, multiple sutures rather than a single one are preferable to alleviate the purse-string effect. In both methods, the surgeons must be careful not to tighten the sutures. In our case, pericardial patch repair was straightforward because the affected lesion was restricted to a clear zone. However, operative procedures are complicated when leaflet destruction extends to the rough zone and first chordae. Therefore, reconstruction of the leaflet and subvalvular apparatus using the pericardium and artificial chordae may be required. Ito et al. reported the reconstruction of the leaflet and chordae with one piece of autologous pericardium for an extensively destructive valve [6]. Hosoba et al. reported excellent midterm results when the affected lesion is restricted to a commissural leaflet or lesions are present in one or two segments [7].

Another concern is the necessity of pretreatment of the autologous pericardium for repair. Surgeons usually have the following options: a fresh pericardium and a glutaraldehyde-treated pericardium. However, several controversies exist regarding shrinkage, calcification, and durability [3,5]. Shomura et al. reported that the glutaraldehyde-treated autologous pericardium has good durability [8]. Although the cohort was not restricted to patients with IE, Fukunaga reported that augmentation using glutaraldehyde-treated pericardium in mitral repair is a risk factor for reoperations at five and 10 years [9]. In contrast, Quinn et al. showed that fresh autologous pericardium has excellent long-term valve function, durability, calcification, and stiffness [10]. The concerns regarding pericardial patch repair include infection, retraction, and calcification, all of which can lead to regurgitation and stenosis. Quinn et al. demonstrated the classification of valve assessments using scores for patch pliability and calcification [10]. Their classifications are represented by numbers and objectives. They revealed that the score did not change over the long term, suggesting that fresh autologous pericardium is a durable material that resists calcification and maintains pliability.

Another concern was the timing of the surgery. Recent studies have shown better outcomes with early surgery (within three days of brain infarction) than delayed surgery [1]. However, in the current case, we performed the surgery six days after the brain infarction, which resulted in no exacerbation. Cardiac surgery using a CPB with systemic heparinization can potentially cause cerebral hemorrhage and infarction. In particular, patients with a recent cerebral infarction may be vulnerable to hemorrhage, which may be critical. Timely surgery and antibiotics administered according to the antimicrobial susceptibility of the bacteria are essential in improving outcomes. The reconstruction of a defective valve using autologous tissue is a good option for the patient complicated with recent brain infarction.

## Conclusions

Mitral valve repair using autologous pericardium is a good option for leaflet perforation due to IE. This is especially preferred for patients with underlying conditions, such as dialysis, steroids, and immunosuppressive drugs.

 Timely surgical intervention is critical for the patient suffering from a recent cerebral infarction due to vegetation.
